# Surface Recombination and Space-Charge-Limited Photocurrent-Voltage (PC-V) Measurements in (Cd,Mn)Te Samples–Kinetics of Photocurrent (PC)

**DOI:** 10.3390/s22082941

**Published:** 2022-04-12

**Authors:** Andrzej Mycielski, Dominika M. Kochanowska, Aneta Wardak, Krzysztof Gościński, Michał Szot, Witold Dobrowolski, Gabriela Janusz, Małgorzata Górska, Łukasz Janiak, Wiesław Czarnacki, Łukasz Świderski, Joanna Iwanowska-Hanke, Marek Moszyński

**Affiliations:** 1 Institute of Physics, Polish Academy of Sciences, Aleja Lotników 32/46, 02-668 Warsaw, Poland; dmkoch@ifpan.edu.pl (D.M.K.); wardak@ifpan.edu.pl (A.W.); kgosc@ifpan.edu.pl (K.G.); szot@ifpan.edu.pl (M.S.); dobro@ifpan.edu.pl (W.D.); gjanusz@ifpan.edu.pl (G.J.); gorska@ifpan.edu.pl (M.G.); 2 International Research Centre MagTop, Institute of Physics, Polish Academy of Sciences, Aleja Lotników 32/46, 02-668 Warsaw, Poland; 3 National Centre for Nuclear Research, Andrzeja Sołtana 7, 05-400 Otwock, Poland; lukasz.janiak@ncbj.gov.pl (Ł.J.); wieslaw.czarnacki@ncbj.gov.pl (W.C.); lukasz.swiderski@ncbj.gov.pl (Ł.Ś.); joanna.iwanowska@ncbj.gov.pl (J.I.-H.); marek.moszynski@ncbj.gov.pl (M.M.)

**Keywords:** CdMnTe, surface defects, etching, carrier mobility-lifetime product

## Abstract

Photocurrent-voltage characteristic (PC-V) is a method of determining the critical parameter in X-ray and gamma-ray detector plates, i.e., the carrier mobility-lifetime product, *μτ*. We show for the (Cd,Mn)Te samples that the measurement results depend strongly on the surface treatment and the space charge distribution. The PC-V characteristics obtained for *ħω* > *E_g_* and *ħω* ~ *E_g_* indicated that etching with 20% HCl caused an appearance of a significant concentration of very shallow surface traps at the (Cd,Mn)Te sample surface. These traps seriously changed the results of measurements of PC-V characteristics and PC kinetics. We also noticed a small contribution of holes to photoconductivity in the PC kinetics. The measurements of PC-V characteristics for *ħω* > *E_g_* may test the detector plate surface quality.

## 1. Introduction

(Cd,Mn)Te is a wide bandgap semiconducting compound. We want to make it competitive with commonly used CdTe and (Cd,Zn)Te compounds for X-ray and gamma-ray detectors. The attained values of the resistivity, *ρ*~10^10^ Ω cm, and the *μτ* product, *μτ* > 10^−3^ cm^2^V^−1^, make us hope for the commercial use of the material. A review of (Cd,Mn)Te studies as material for those detectors may be found in Reference [1]. One of the critical parameters is the *μ_e_τ_e_* product, where *μ_e_* is the carrier mobility (here electrons) and *τ_e_* is the carrier lifetime (here also electrons). It is not easy to attain a large *μ_e_τ_e_* parameter (>5 × 10^−3^ cm^2^V^−1^), uniform in the whole volume of the sample with an area of 20 × 20 mm^2^ or more. Usually, the *μ_e_τ_e_* parameter is determined by illuminating the sample with alpha particles or gamma rays and investigating the charge collection efficiency (CCE). CCE is defined as the fraction of injected charges that is collected in the external circuit. For alpha particles with energy of 5.5 MeV, the penetration depth is about 20 µm. Concerning the typical sample thickness (~2000–3000 µm), it is like a surface effect. The penetration is much deeper for the gamma radiation (Am-241 59.5 keV). The incident gamma radiation with energy of 60 keV is absorbed in 90% at a distance of about 600 µm; it is ~20% of the sample thickness. The CCE value may be obtained by fitting the experimental PC-V data with the Hecht–Many function [2,3], where the fitting parameters are *μ_e_τ_e_* and the surface recombination velocity, *s_e_*.

The Hecht–Many model assumes no space charge in the sample in stationary conditions. Therefore, the internal electric field is uniform: *E = VL*^−1^, where *E* is the field, *V* is the applied voltage, and *L* is the sample thickness. The model also assumes that the parameters *µ_e_* and *τ_e_* are uniform in the sample volume. This means that *μ_e_* ≠ *μ_e_*(*a*) and *τ_e_* ≠ *τ_e_*(*a*), where *a* is distance from the illuminated cathode to the anode. Next, in this model an electric charge is generated just below the sample surface at a distance *δ* much smaller than the sample thickness, *L*. For (Cd,Zn)Te, Cui et al. [4,5] have shown that the *μ_e_τ_e_* product may be determined by measuring the photocurrent as a function of the applied voltage (PC-V). The energy of the light used for the electron injection was either higher or smaller than the energy gap, *E_g_*. The experiments indicated that the results strongly depended on the surface conditions and the surface processing methods. The authors recall similar observations of the impact of surface conditions on the carrier transport measurements in HgI_2_ [6,7,8].

The aim of our study was to perform comparable measurements for (Cd,Mn)Te by using the PC-V technique and different methods of sample surface preparation. Similar experimental and theoretical studies were performed for (Cd,Zn)Te [9,10,11,12,13]. Shen et al. [14] showed interesting results of processing the (Cd,Mn)Te surfaces. The main conclusion of that study was that the final etching with 5% HCl created the smoothest surface with the average value of the surface roughness, *R_a_*, 0.84 nm. We decided to test Shen’s method. We etched the polished surfaces of the (Cd,Mn)Te samples with HCl, changing the concentration of the solution.

In the next sections, we present results of measurements of the PC-V characteristics. The results obtained by using LED light were compared with the measurements performed by using the gamma rays (Am-241 59.5 keV). We show that the results of the PC-V investigation are significantly different for different methods of the sample surface preparation. Finally, we present the PC-V characteristics for the excitation by the light with the energy above *E_g_* and slightly below *E_g_*.

## 2. Materials and Methods

The crystals of Cd_0.95_Mn_0.05_Te:In ([In] ≈ 1 × 10^17^ cm^−3^) were prepared by the low-pressure Bridgman (LPB) method in the vertical configuration. The diameters of the crystals were 2 or 3 inches and the weight 500 g and 1500 g, respectively. Usually a few twins which pass from the bottom to the top of the crystal are visible (see, e.g., Figure 22 in Reference [1]). Single-crystal blocks allowed us to obtain monocrystalline plates with dimensions of 20 × 20 mm^2^, 30 × 30 mm^2^, or more. The concentration of Te inclusions is ≤5 × 10^3^ cm^−3^. To obtain semi-insulating materials with *ρ*~10^10^ Ω cm, the crystals were doped with indium. To obtain deep donors Te_Cd_^2+^ [15,16], tellurium was added in proportion 50–100 mg Te per 100 g of material. The crystallization speed was 0.5–1.0 mmh^−1^. Before cutting, the crystals were etched with the Durose etchant [17,18] to reveal twins and grain boundaries. Durose etchant reveals the polarity of the CdTe plate cut along the (111) plane, i.e., the twin plane. Then, the Cd-face is (111)-oriented and is named A-face, and the Te-face is $\left(\bar{1}\bar{1}\bar{1}\right)$-oriented and is named B-face. The etched Cd-face is matt black and the Te-face is shiny. The (Cd,Mn)Te crystal usually consists of a few, differently oriented monocrystalline blocks and a few twins. Basing on the shade of gray of differently oriented grains in the (Cd,Mn)Te crystal, one can clearly determine the position of grain boundaries and twin planes. Then, the samples were cut parallel to the twin planes (111). The samples were initially ground and etched with Durose etchant to determine the planes A (cadmium) and B (tellurium). After that, the samples were mechanically ground by using a water suspension of 4.5 μm SiC powder, and mechano-chemically polished in 2% bromine-methanol solution. The mechano-chemical polishing of the samples was followed by etching with HCl acid.

Min Shen et al. [14] described interesting methods of preparing surfaces of (Cd,Mn)Te samples for depositing contacts. The results inspired us to use HCl in the procedure of the sample surface preparation. The last stage of the authors’ method is chemical polishing (CP) with 5% HCl. We prepared three sets of samples, the surfaces (A and B) of which were treated with differently concentrated HCl, namely: 10%, 15%, or 20% for 10–20 s. In the next sections we will show that these three sets of samples can be divided into two groups depending on the observed surface effects. The first group of samples was etched with 10% or 15% HCl. We do not observe any effects related to the generation of surface traps for this group. The second set of samples was etched with 20% HCl. The density of generated surface traps is significant for this group.

Onto both types of surfaces, A and B, thin contact layers with thicknesses ~30 nm were deposited by using the sputtering method. For this purpose, we used two types of metal alloys: Au/Pd or Pt/Pd, where the proportion of Au or Pt to Pd was 80:20. Palladium added to Au or Pt target increases adhesion. The monocrystalline samples had dimensions of 10 × 10 mm^2^ or 20 × 20 mm^2^ and thicknesses roughly of 1–3 mm. In the center of the sample surface the metal contact layers (anode and cathode) had diameters *φ* = 5 mm for samples with dimensions 10 × 10 mm^2^ and *φ* = 8 mm for samples with dimensions 20 × 20 mm^2^. On the surfaces close to the sample edges, rings, whose role was to insulate the current between the anode and the cathode from the leakage current on the sidewalls of the sample, were deposited by sputtering. For PC-V measurements, the light sources were light-emitting diodes (LEDs), and the light passed through interference filters (IF) to reduce the width of a spectral range of the exciting radiation. The Full Width at Half Maxima (FWHM) of the IF will be shown in Section 3.1. The central part of the cathode was illuminated through an aperture with *d* = 3 mm for samples with dimensions 10 × 10 mm^2^ and *d* = 5 mm for samples with dimensions 20 × 20 mm^2^. The incident light power in μW, converted to eV/s and divided by the photon energy, enabled us to determine the number of incident photons. 

Each photon, reaching the sample surface through the metal contact, especially with the energy *ħω* > *E_g_*, creates an electron-hole pair. We believe that for thin (approximately 30 nm) metal contacts, the loss of light intensity is mainly due to reflection. We estimate that at least 30% photon flux reaches the sample. The energy gap, *E_g_*, of Cd_0.95_Mn_0.05_Te crystals is 1.575 eV at room temperature. For *ħω* greater than *E_g_* (e.g., *E_g_* + 30 meV) the absorption coefficient in our samples is about 2 × 10^4^ cm^−1^ [19,20]. The radiation is absorbed at a distance from the surface smaller than 1 µm. Holes quickly return to the cathode and electrons conduct the electric current. 

So, the current measured in the PC-V characteristic is an electron current. The PC-V characteristics were measured at a constant illumination of the middle part of the cathode by increasing the bias voltage. Kinetics of photocurrent was measured at a selected constant bias voltage by applying a nearly rectangular light pulse (pulse rise time ~0.5 ms). As will be shown in Section 3.2, the increase and stabilization of the PC signal after sample illumination, and the extinction of the PC signal after turning off the light source last longer than 1 s. Therefore, we did not use a chopper in the experiments of photocurrent kinetics. We excited the samples with photon energies *ħω* ~ *E_g_* and *ħω* > *E_g_*, i.e., *ħω = E_g_ + x*, where *x* changed from 15 meV to 76 meV and *E_g_* = 1.575 eV.

The experiments of PC-V characteristics with the use of LEDs and photocurrent kinetics were performed with the use of the following equipment:

- The PC-V measurements were carried out by using the samples with rings. The grounded cathode was illuminated by LED. The cathode and ring on the cathode side were biased with the same negative voltage, and the anode and the ring on the anode side were biased with the same positive voltage. Two Keithley 6517B Electrometers were used to apply the bias voltage and to measure the current between cathode and anode and the current between rings on the cathode and anode sides.
- Illuminating Thorlabs LEDs were powered by the Keithley 6220 Precision Current Source. We used six types of LEDs, the maximum light intensities of which are at energies: 1.434 eV, 1.507 eV, 1.556 eV, 1.590 eV, 1.602 eV and 1.656 eV. The FWHM of the LED emitted spectra is approximately 58 meV. The interference filters we used are characterized by maximum transmissions at 1.461 eV, 1.509 eV, 1.556 eV, 1.608 eV and 1.651 eV. The FWHM of IF are from 16 to 23 meV. In our measurements in the energy range *E_g_* − 15 meV > *ħω > E_g_* + 15 meV a small value of IF FWHM is negligible but in measurements at *ħω ~ E_g_* the IF FWHM may be misleading due to a sharp change of the absorption coefficient in this spectral range.
- The LED light passing through IF was uncovered and covered by the Thorlabs SC10 Shutter Controller. The shutter open and close time was about 0.5 ms. Radiation power was measured by the Thorlabs PM100D Optical Power Meter.
- PC kinetics was registered by the Tektronix MSO 54 Oscilloscope by using the pre-amplifier from the Keithley 6517B Electrometer.
- The PC-V characteristics were measured at room temperature 294 K (21 °C) and at a higher temperature of 306 K (33 °C) by using a slightly heated sample holder.

We also made measurements as follows:

- The room-temperature current-voltage (I-V) characteristic was measured at a voltage up to ±700 V by using the Keithley 6517B voltage source.
- The resistivity map was obtained by a contactless method, i.e., the Time-Dependent Charge Measurement (TDCM) method [21], with the use of the EU-*ρ-μτ*-SCAN apparatus. In the TDCM method the sample is placed into a capacitor as a lossy dielectric substance and the resistivity is evaluated by measuring a time-dependent charge transient observed after application of a voltage step. The measurement is non-contacting, i.e., it avoids the problems connected with the fabrication of ohmic contacts [22].
- Measurement of the absorption coefficient (absorption edge) was performed at room temperature on a 100 µm thick sample by using the Fluorolog 3 spectrometer equipped with a photomultiplier as a detector.
- The measurements of the gamma-ray response current were made by using the Am-241 gamma source, the activity of which was 925 MBq (single-encapsulated stainless steel source). A small lead collimator with a hole diameter of 3 mm and a length of 3 cm was used. The current flowing through the sample under X-ray irradiation was measured with a digital multimeter Keysight 2901A.
- Alpha particle energy spectra for (Cd,Mn)Te samples were examined with a spectrometric system. This system consisted of a charge preamplifier, a spectrometric amplifier and a multichannel analyzer. Alpha particle energy was 5.5 MeV.
- The measurement of the X-ray spectrum centroid as a function of the applied voltage was made by using the Spectroscopic Pixel Mapping apparatus by Eurorad. The activity of the Co-57 source used by us was 398 kBq. The sample was polarized by a negative high voltage. The spectrum was performed via a multichannel analyzer, which was connected to the amplifier.
- In the experiments with the use of alpha particles and X- and gamma rays, the application of rings was eliminated. The incident particles or radiation passed through a collimator and reached the sample from the cathode side.

## 3. Results and Discussion

### 3.1. Photocurrent-Voltage Characteristic

**Figure 1:**
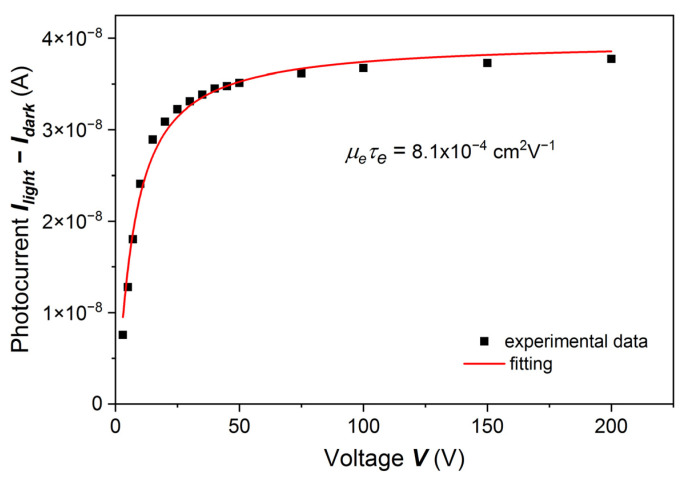
Room-temperature electron photocurrent as a function of a bias voltage for the Cd_0.95_Mn_0.05_Te: In sample etched with 10% HCl. The illuminating light energy was *ħω* *=* 1.590 eV *(E**_g_** +* 15 meV). The solid line is a fit with the Hecht equation. The value of the *µ_e_τ_e_* parameter is 8.1 × 10^−4^ cm^2^V^−1^ with a standard error of 0.47 × 10^−4^ cm^2^V^−1^. The contacts were sputtered ~30 nm thick Au/Pd layers.

In the first set of Cd_0.95_Mn_0.05_Te:In samples, the sample surfaces were etched with 10% or 15% HCl. The PC-V characteristic of one of the samples is shown in Figure 1. We assume that *μ_e_* ≠ *μ_e_(a)*, *τ_e_* ≠ *τ_e_(a)* and *δ* ≪ *L*, as was mentioned in Section 1. The experimental points were fitted with the Hecht formula:

(1) $$I=\frac{I_{0}\mu_{e}\tau_{e}E}{L}\;\left[1-\exp\left(-\frac{L}{\mu_{e}\tau_{e}E}\right)\right],\;$$

where *I*_0_ is the saturation photocurrent, *μ_e_* is the electron mobility, *τ_e_* is the electron lifetime, *E* is the electric field intensity, and *L* is the sample thickness. The fitting parameters were *µ_e_τ_e_* and *I*_0_. The agreement of the model with the experimental data was checked by R-squared test. The *µ_e_τ_e_* value obtained from the fit was about 8.1 × 10^−4^ cm^2^V^−1^, and *I*_0_ = 3.9 × 10^−8^ A. The energy of the illuminating LED light was slightly above the energy gap, *ħω* = 1.590 eV (*E_g_ +* 15 meV). The absorption coefficient for this light energy is *α* > 10^4^ cm^−1^, so the light is absorbed at a distance from the sample surface smaller than 1 µm.

**Figure 2:**
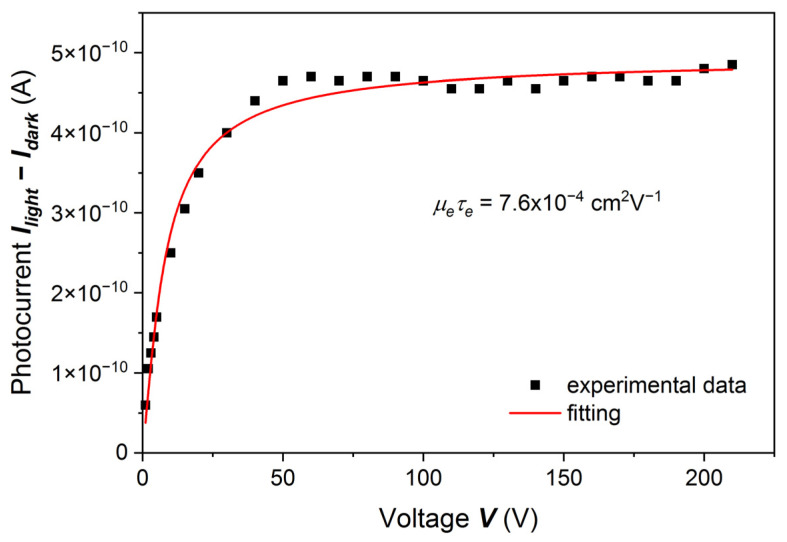
Room-temperature Am-241 gamma-ray response current for the (Cd,Mn)Te:In sample measured in Figure 1. Gamma radiation energy is 59.5 keV. The solid line is a fit with the Hecht equation, *μ_e_τ_e_* = 7.6 × 10^−4^ cm^2^V^−1^ with a standard error of 0.44 × 10^−4^ cm^2^V^−1^.

For the same sample, the results of which are shown in Figure 1, we measured the gamma-ray-induced current by using 59.5 keV gamma rays from an Am-241 source. The Hecht formula may be applied when the charge carriers are generated just below the surface. However, we were curious about whether we would get a fundamentally different result by excitation with X-rays from the Am-241 source. The results fitted with the Hecht formula are shown in Figure 2. The fitted curve has the same shape as the curve obtained from the PC-V measurement, and the fitting parameters are *µ_e_τ_e_* = 7.6 × 10^−4^ cm^2^V^–1^, *I_0_* = 0.49 × 10^−9^ A.

Moreover, we measured the CCE of this (Cd,Mn)Te sample by using alpha particles. The *µ_e_τ_e_* value obtained from the Hecht equation (Equation (1)) for alpha particles is *µ_e_τ_e_ =* 3.1 × 10^−4^ cm^2^V^–1^ with a standard error of 0.15 × 10^−4^ cm^2^V^–1^. In the experiment with the use of alpha particles the background noise was quite high. Nevertheless, we were able to register the X-ray spectrum centroid as a function of the applied voltage. 

**Figure 3:**
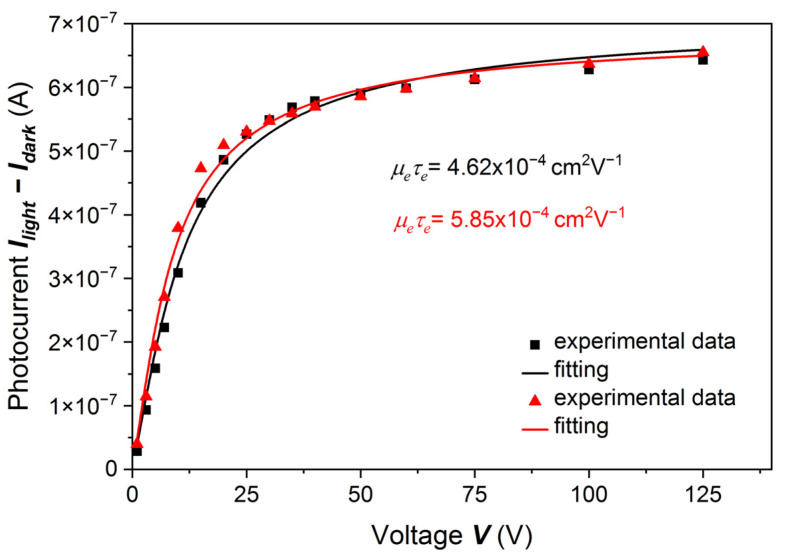
PC-V characteristic for two opposite surfaces (A and B) of a (Cd,Mn)Te:In sample etched with 15% HCl. Red and black symbols and curves represent results and fittings obtained for the Cd side (A) and Te side (B), respectively. The fitting was made with the Hecht equation. The standard error of *µ_e_τ_e_* is 0.3 × 10^−4^ cm^2^V^−1^. The contacts on both surfaces of the sample were sputtered Au/Pd layers with thicknesses ~30 nm.

In References [4,5], the authors observed a considerable difference in the PC-V characteristics measured on A and B surfaces of the investigated samples. We also measured PC-V characteristics of both surfaces of our samples. The results are shown in Figure 3. Results for both surfaces were fitted with the Hecht formula, and the fitting parameters were slightly different. For the A surface we obtained *µ_e_τ_e_* = 5.85 × 10^−4^ cm^2^V^−1^, *I*_0_ = 6.9 × 10^−7^ A, and for B *µ_e_τ_e_* = 4.62 × 10^−4^ cm^2^V^−1^, *I*_0_ = 7.1 × 10^−7^ A.

Comparing the results shown in Figure 1, Figure 2 and Figure 3, we concluded that for the first group of samples, i.e., when the sample surfaces were treated by less concentrated HCl, namely 10% or 15%, the results of both experiments, i.e., photocurrent measurements with the use of LED and Am-241 radiation sources, can be well described by the Hecht formula. Below we will discuss certain shortcomings of this method.

**Figure 4:**
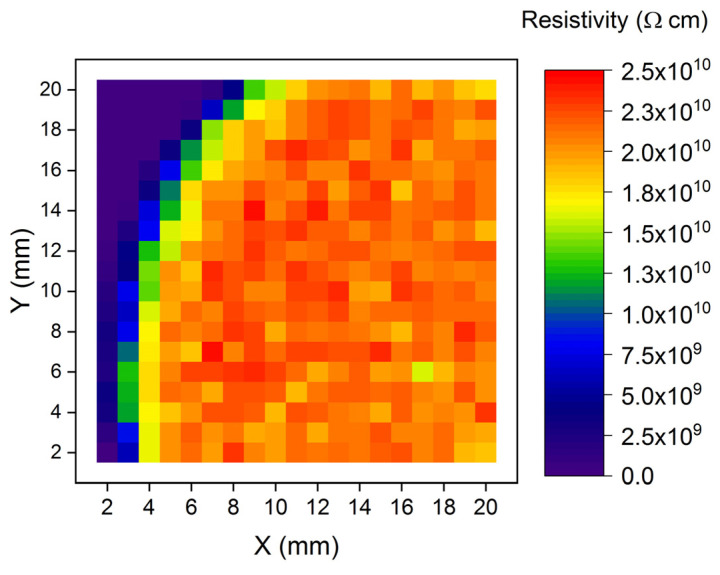
Resistivity map of a (Cd,Mn)Te:In sample measured by the EU-*ρ*-*μτ*-SCAN apparatus. The upper left corner is the border of the sample (and of the crystal). The side surface leakage current influences the measured sample resistivity up to about 3 mm from the side.

Before the PC-V characteristics, resistivity maps of all investigated samples were obtained. A typical result is shown in Figure 4. Let us pay attention to the upper left corner of the map, the sample border (and the crystal border). We see that the leakage side current may influence the measurement result up to about 3 mm from the side. Therefore, in measurements of PC-V and I-V we used rings close to the sample sides.

Semi-insulating samples, chosen for measurements of PC-V characteristics and PC kinetics, had resistivities with average values from 5 × 10^9^ Ω cm to 2 × 10^10^ Ω cm.

**Figure 5:**
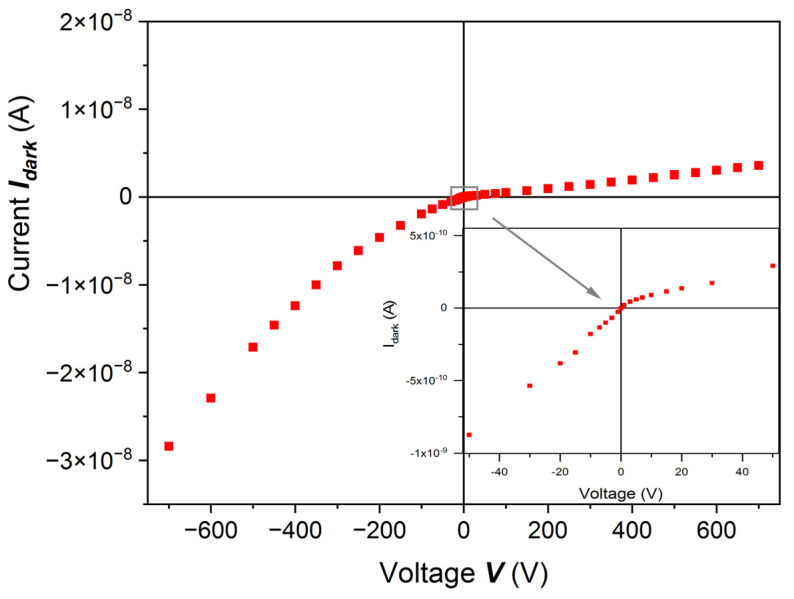
Room-temperature dark I-V characteristic for a (Cd,Mn)Te:In sample cut parallel to the (111) plane. After preliminary preparation, the sample was etched with 20% HCl. The contacts were sputtered Pt/Pd layers. The current is much lower when the anode is on the cadmium surface (A), especially at high bias voltage.

As was mentioned in Section 2, the samples were cut out of the crystals parallel to the (111) plane. Metal contact layers, deposited onto polished and etched surfaces, had thicknesses ~30 nm. In the I-V measurements, we noticed that when the anode (+) was on the A (cadmium) side, the current had a lower value than when the current was applied in the opposite direction, especially for the high voltage. This is due to the non-ohmic contacts. A typical I-V characteristic is shown in Figure 5. The right side of the plot shows the case when the anode is on the A (cadmium) side. This effect was checked on many samples with surfaces prepared by different methods. Experimentalists may pay attention to that.

**Figure 6:**
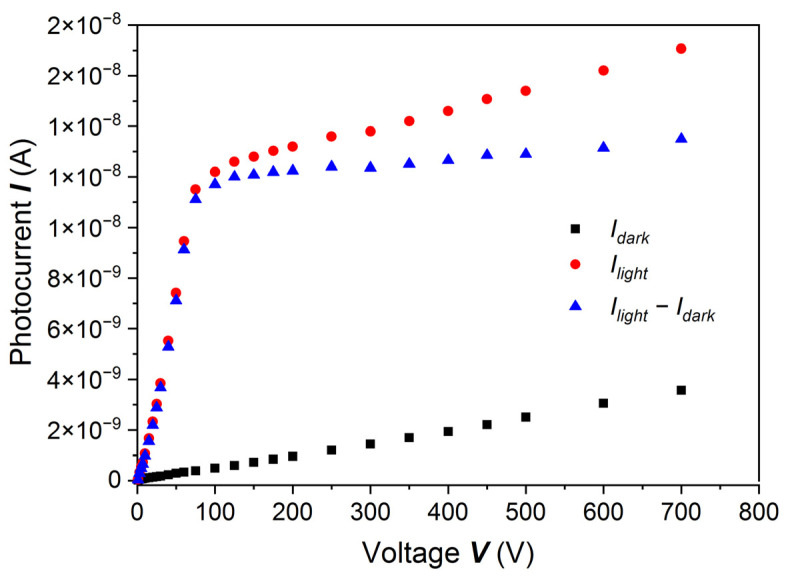
PC-V characteristics for a (Cd,Mn)Te:In sample etched with 20% HCl. At voltages up to ~75 V the curve does not look like the exponential Hecht–Many function, rather like a straight line or power-like function. The illumination photons energy was very close to *E_g_*, i.e., *ħω* = 1.590 eV = *E_g_* + 15 meV.

PC-V characteristics measured for the second group of samples treated with 20% HCl are shown in Figure 6. Let us look at the first part of the PC-V curve in the range 1–75 V. It is not concave, like in the Hecht or Hecht–Many exponential formula, but slightly convex. The sample was illuminated by photons with energies of 1.590 eV, slightly above the *E_g_* (*E_g_* = 1.575 eV). This result caused us to start a series of investigations on (Cd,Mn)Te samples with surfaces prepared by etching with 20% HCl. We performed experiments with the use of interference filters, in the energy range *ħω* > *E_g_* and *ħω* ~ *E_g_*, and for different light intensities. These results will be shown below.

**Figure 7:**
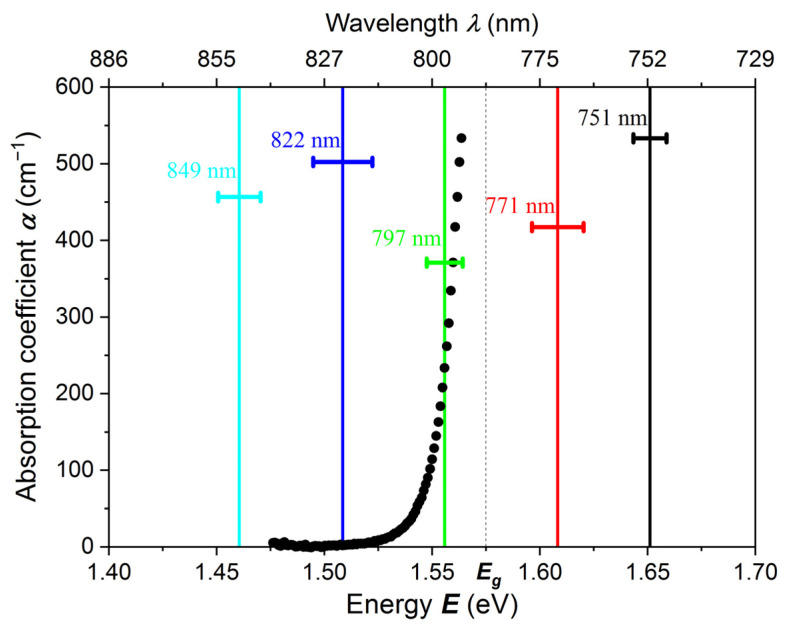
The tail of the absorption edge as a function of light wavelength and photon energy for a Cd_0.95_Mn_0.05_Te:In sample with a thickness of 100 μm. To obtain the PC-V characteristic the light was emitted by LEDs and passed through interference filters. Half widths of some filters are marked.

In Figure 7 we show the tail of the absorption edge for one of the Cd_0.95_Mn_0.05_Te:In samples. For absorption measurement we used a sample prepared by mechano-chemical polishing with 2% bromine-methanol solution. The sample thickness was *L* = 100 μm and the reflection *R* = 0.18 (18%). In Figure 7 there are also marked energies, wavelengths and the bandwidths of the light passing through the interference filters. For example, for the interference filter 797 nm (1.556 eV), for which the absorption coefficient is *α* ~ 200 cm^−1^, about 90% of the radiation is absorbed at a distance of ~100 µm.

**Figure 8:**
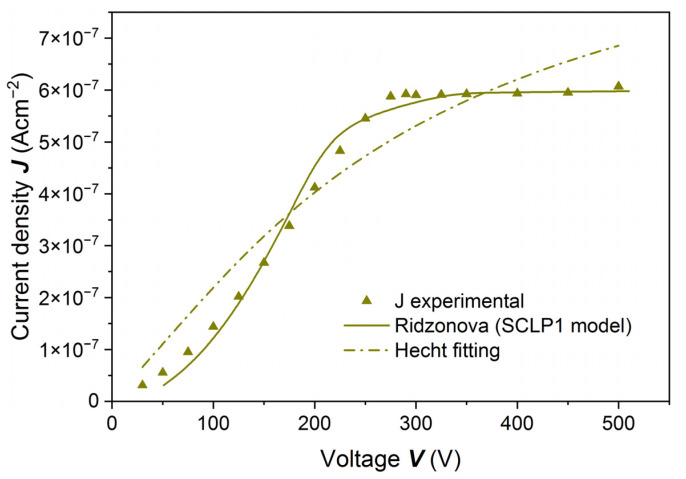
Current density *J* as a function of voltage *V* at the mean photon flux *F* = 8.49 × 10^12^ cm^−2^s^−1^ measured on a (Cd,Mn)Te:In sample etched with 20% HCl. The excitation energy was equal to *ħω* = 1.651 eV = *E_g_* + 76 meV. The dashed line is a fit with the Hecht formula, solid line is a fit with the Ridzonova et al. model [13].

To interpret the surprising results for the sample etched with 20% HCl, shown in Figure 6, we measured the PC-V characteristics by using the photon energy *ħω =* 1.651 eV (*E_g_ +* 76 meV), for which the absorption coefficient *α* ~ 2 × 10^4^ cm^−1^ [19]. By exciting the sample with photons with such energy, one generates charge carriers at a distance smaller than 1 µm from the sample surface. In Figure 8 current density, *J*, is shown as a function of voltage, *V*. It was measured at the mean photon flux *F* = 8.49 × 10^12^ cm^−2^s^−1^. With increasing voltage, the current density increases at first as a power function, then reaches saturation and does not change up to the highest applied voltage. The experimental results shown in Figure 8 could not be fitted with the Hecht formula, and even less with the Hecht–Many formula:

(2) $$I=\frac{I_{0}}{1+\frac{s_{e}}{\mu_{e\;}E}}\;\frac{\mu_{e}\tau_{e}E}{L}\;\left[1-\exp\left(-\frac{L}{\mu_{e}\tau_{e}E}\right)\right],$$

where *s_e_* is the surface recombination velocity.

**Figure 9:**
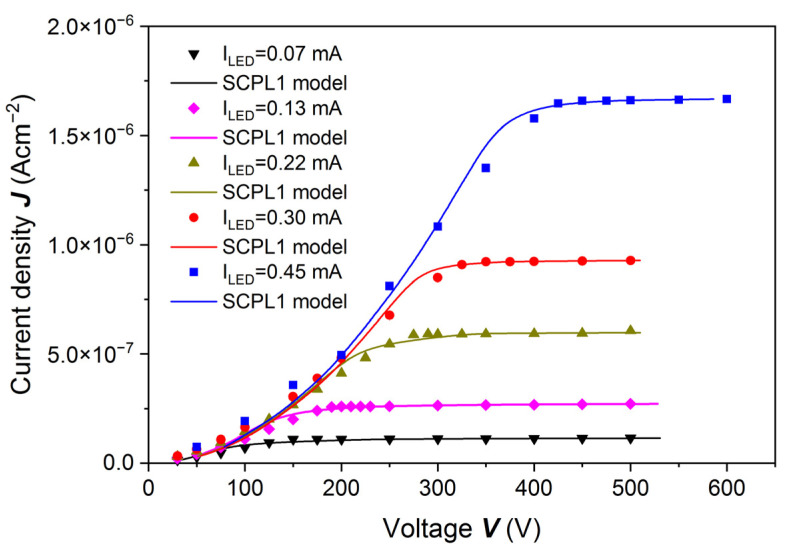
Current density *J* as a function of voltage *V* obtained for a (Cd,Mn)Te:In sample etched with 20% HCl. PC-V characteristics were measured at different photon fluxes. The excitation energy was equal to *ħω* = 1.651 eV = *E_g_* + 76 meV. For the lowest photon flux, *F* = 1.39 × 10^12^ cm^−2^s^−1^, the saturation is reached at 150 V. For the highest photon flux, *F* = 2.36 × 10^13^ cm^−2^s^−1^, the saturation is reached at 450 V. Solid lines are fitted to the Ridzonova et al. model.

We measured the PC-V characteristics at different photon fluxes for the same sample, the results of which are shown in Figure 6 and Figure 8. The results of calculated current densities as a function of voltage are shown in Figure 9. Solid lines in Figure 9 are fitted to the Ridzonova et al. model [13]. The fitting parameters are listed in Table 1. For the lowest photon flux, *F* = 1.39 × 10^12^ cm^−2^s^−1^, the photocurrent saturation is reached at 150 V. For the highest photon flux, *F* = 2.36 × 10^13^ cm^−2^s^−1^, the saturation is reached at 450 V.

To interpret the results shown in Figure 9, we followed the model proposed by Ridzonova et al. [13] (Equation (9) in that cited paper):

(3) V=ε0 εrμe θ3J {[(seJμe(F*e−J))2+2JLε0 εrμeθ]32−(seJμe(F*e−J))3},

where *J* represents the current density, ε_0_, ε_r_, *V*, F*, and *e* are the vacuum and the relative permittivity, the applied bias voltage, the photon flux which reaches the sample passing the metal layer and the elementary charge, respectively. Parameter *θ* is defined as follows:

(4) $$\theta =\frac{n}{n+n_{t}}\;,$$

where *n* is the free electron density, and *n_t_* is the trapped electron density. Usually *n_t_* >> *n*, so $\theta =\frac{n}{n_{t}}$. In this model, the fitting parameters were *θ* and *s_e_*. The F* value was determined from the saturation of photocurrent versus voltage [13].

The model considers surface effects and the non-uniform electric field distribution in the sample, especially in the vicinity of the cathode. Fits of the experimental data with the model from Reference [13] are shown in Figure 9.

**Figure 10:**
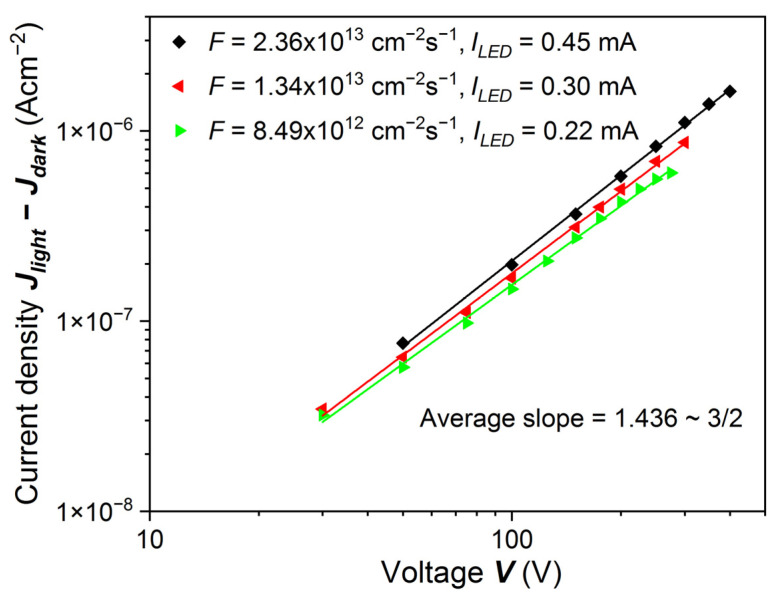
PC-V characteristics from Figure 9 in logarithmic scale. The excitation energy was equal to *ħω* = 1.651 eV = *E_g_* + 76 meV. Photocurrent increased with the bias voltage roughly like *V*^3/2^. When the current is inversely proportional to the cross section for Coulomb trapping, it should depend on the electric field like *E*^3/2^ [23,24,25,26].

We can see that for the sample etched with 20% HCl, the photocurrent measured at different photon fluxes changes exponentially with applied bias voltage, with the exponent close to 3/2, which is shown in Figure 10. Such behavior may be expected for traps generating a “Coulomb-attractive” potential. This effect is similar to the Poole–Frenkel model [23,24] analyzed in Reference [25]. In that paper, the de-trapping mechanism is described by the relation *E*^3/2^, following the change of the capture cross section proportional to *E*^−3/2^. Such a relationship was obtained in Reference [26], where in lithium-doped germanium detectors a decrease of the trapping cross section as a function of electric field was observed (see Figure 17 in Reference [26]). The results and their interpretation indicate quite deep traps.

For a sample etched with 20% HCl, the results of which are indicated in Figure 6, Figure 8, Figure 9 and Figure 10, we registered X-ray spectra using Co-57 source. According to our interpretation, saying that a treatment of (Cd,Mn)Te sample with 20% HCl causes a formation of surface traps, we cannot expect sharp peaks in the detected spectra. Instead of peaks we observed broad maxima shifting with the applied voltage up to 450 V. The position of a maximum is stabilized in a voltage region from 500 V to 700 V.

**Figure 11:**
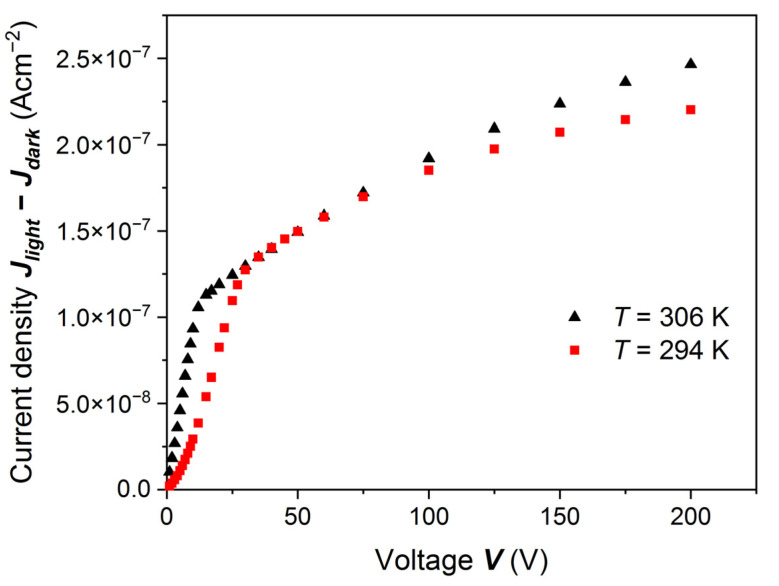
PC-V characteristics measured at two slightly different temperatures. The results were obtained on a (Cd,Mn)Te:In sample etched with 20% HCl but different from the one whose results were presented in Figure 6, Figure 8, Figure 9 and Figure 10. The excitation energy was equal to *ħω* = 1.608 eV = *E_g_* + 33 meV. Below 30 V at 294 K, the dependence is power like. It changes shape after raising temperature up to 306 K (by about 4%) and resembles the Hecht–Many function. The conducting electrons may appear due to thermal ionization of surface trapping. In this sample deep traps must also exist, e.g., tellurium antisites Te_Cd_^2+^. Deep traps can be emptied by the application of higher voltages. Thus, in a voltage range higher than 50 V the increase of the PC-V signal is noticed.

In order to explain the results presented in Figure 6, Figure 8, Figure 9 and Figure 10, we measured PC-V characteristics for another (Cd,Mn)Te sample at two slightly different temperatures: 294 K and 306 K. The result is shown in Figure 11. The small temperature change (~4%) caused an evident change in the measured PC-V curve. The dependence changed from a power function to a nearly exponential function at the low voltage, resembling the Hecht–Many function. Because of the presented results, we believe that we observe very shallowly localized (i.e., localized close to the sample surface) traps capturing electrons moving from the cathode, where they are generated by light. The increasing voltage causes de-trapping, and at the higher voltage the PC-V curve saturates. Figure 11 indicates that in a low voltage region a temperature change from 294 K to 306 K causes an increase of the PC-V signal by a factor of 2.5. We estimated that the ionization energy of these surface traps is approximately 0.6 eV below the conduction band. It is valid for semiconductors partially compensated [27]. Their ionization at low voltage may be similar to the Poole–Frenkel effect. Moreover, for (Cd,Mn)Te crystals, the measurements of the Pockels effect and Pockels E-field profiling [28,29,30] indicated at the high voltage the non-uniform distribution of the electric field in the sample up to about 200 μm from the cathode. Therefore, we cannot consider the electric field inside the sample, *E* as *VL*^−1^, because the field is not uniform. We can only take into consideration the applied voltage, as was done in Reference [13].

Due to the fact that from the Ridzonova model [13] we could not determine the *μ_e_τ_e_* value, we made an attempt to roughly estimate this value. We used the relation:

(5) $$J=e\;N_{e}\;\mu_{e}\frac{V}{L}\;,$$

where *N_e_* is the free electron concentration. Here we assume *μ_e_* = 800 cm^2^V^−1^s^−1^ [31,32,33]. From this relation and the PC-V saturation current, we obtained the free electron concentration, *N_e_*, given in Table 1.

In Figure 9, we see for each PC-V curve a definite knee, above which the current saturates. Therefore, we believe that all electrons generated by light reach the anode for the bias voltage, *V*, corresponding to the knee. That would mean that the mean drift time, *τ_dr_*, corresponds to the electron lifetime, *τ_e_* (*τ_d_*_r_ ≈ *τ_e_*).

We are aware of the possible non-uniform electric field distribution, especially for high bias voltage and high photon flux. However, we try to estimate the *μ_e_τ_e_* product from the relation:

(6) $$v_{dr}=\mu_{e}\;E=\frac{L}{\tau_{dr}}\;,$$

Then, $L≅\;\mu_{e\;}\tau_{e}\;E$ and $\mu_{e}\;\tau_{e}≅\;\frac{L^{2}}{V}$.

We estimated the *μ_e_τ_e_* value for the lowest photon flux *F* = 1.39 × 10^12^ cm^−2^s^−1^ as approximately 7 × 10^–4^ cm^2^V^–1^. For the highest photon flux *F* = 2.36 × 10^13^ cm^−2^s^−1^ in Figure 9, the knee is at the highest voltage, about 400 V, and the *μ_e_τ_e_* value is three times smaller. We realize that the electric field distribution is not uniform for higher photon fluxes and bias voltages, as was observed by the Pockels effect [28,29,30], and the estimated *μ_e_τ_e_* is several times smaller.

### 3.2. Kinetics of Photocurrent

The kinetics of photocurrent was measured for the sample, the PC-V characteristics of which are presented in Figure 8 and Figure 9. A bias voltage was applied to the sample and a dark current stabilized after some time. Then a shutter was opened, and a nearly rectangular light illumination was applied on the cathode side. The shutter open time was about 0.5 ms. The light passed through an interference filter. The light energies were *ħω* > *E_g_* and *ħω* ~ *E_g_*. Photocurrent was registered as a function of time. Then, a higher bias voltage was applied and the measurement was repeated.

**Figure 12:**
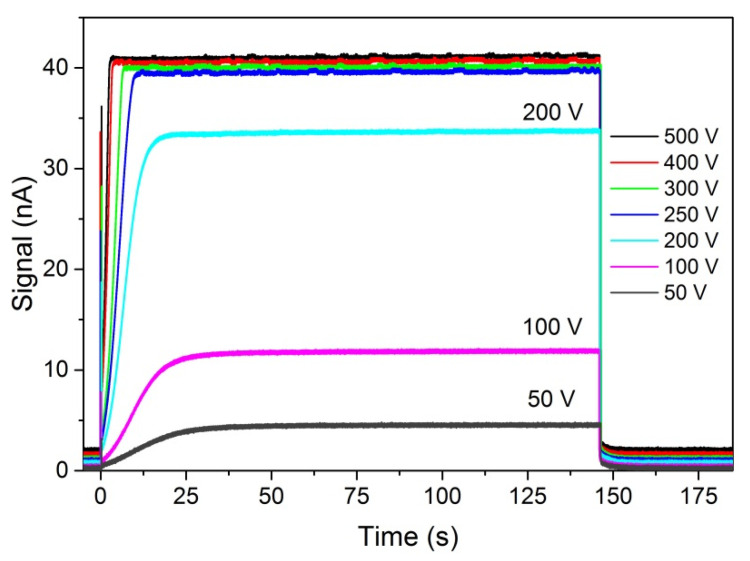
PC kinetics after rapid (~0.5 ms) turning on the illuminating light, *F* = 1.55 × 10^12^ cm^−2^s^−1^, *ħω* *=* 1.608 eV (*E_g_* + 33 meV), at different voltages. Charge carriers are generated at the surface of the sample. The PC time dependence does not change much above 250 V.

The same bias voltage was applied to the electrodes and to the rings at the sample edges for every run. The current was measured with the Keithley 6517B electrometer, displayed on the Tektronix MSO 54 Oscilloscope, and registered. The time base was 180 s. Using stable light with energy 1.608 eV, which is ~33 meV above the energy gap and which enters the sample at a distance smaller than 1 μm, we apply consecutively higher bias voltages to obtain the results shown in Figure 12. The sharp peak at *t* ~ 0 in Figure 12 is probably an artifact from electronics. At the lowest voltage (50 V), the signal rise time till stabilization is about 40–50 s. The signal amplitude at 50 V is about ten times smaller than the one at the highest voltage (500 V). At the highest voltage, the signal rise and stabilization time reduce to ~3 s.

The saturation current values increase with bias voltage like in the spectra shown in Figure 9. However, after shutting (shutter close time ~0.5 ms) the photocurrent decay is fast and does not depend on the bias voltage. Decay time has two components: (1) Very short time, much less than 1 s; (2) time about 5–8 s. The time decay was not analyzed in detail in the present work.

**Figure 13:**
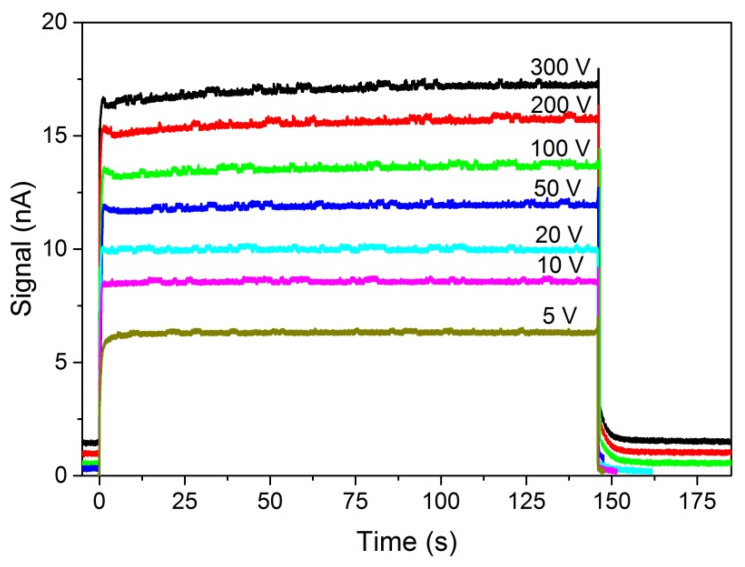
PC kinetics for the sample shown in Figure 12, *F* = 3.83 × 10^12^ cm^−2^s^−1^, *ħω* *=* 1.531 eV (*E_g_* − 44 meV). Charge carriers are generated inside the sample, not only at the surface. The shape of the PC time dependence does not depend on the bias voltage; only the amplitude depends on the bias voltage. The slight rise of the PC signal with time at *V* ≥ 100 V was attributed to the influence of holes.

The kinetics of photocurrent for the photon energy *ħω* = 1.531 eV (*E_g_* − 44 meV) is very different from the one observed when *ħω* > *E_g_* (Figure 12), and is shown in Figure 13. For such photon energy in the tail of the absorption edge, the absorption coefficient is about 20 cm^−1^. The LED light with energy of 1.531 eV is absorbed in 90% at a distance of about 350 μm, which is about 10% of the thickness of this sample (3210 μm). For comparison, Am-241 gamma radiation with energy of 60 keV is absorbed in 90% at a distance of about 600 µm, as was mentioned in Section 1. If defects created by etching the sample surface with 20% HCl are surface defects, we can expect different PC characteristics for *ħω > E_g_* and *ħω < E_g_.* The light of energy *ħω > E_g_* is absorbed close to the sample surface and can interact with surface defects. The light of energy *ħω < E_g_* penetrates the sample deeper and to a lesser extent generates charge carriers near the surface. A similar phenomenon takes place if the sample is illuminated by gamma rays from Am-241 59.5 keV. Therefore, the Am-241 source cannot be used in photocurrent kinetics investigations. In Figure 13, we can see that after opening the shutter, the signal rise time at the lowest voltage (5 V) is about 2 s, and at the highest voltage (300 V) is about 1 s. Decay time, like for *ħω* > *E_g_*, is at first very fast and thereafter several seconds and does not depend on the bias voltage. For *ħω* = 1.531 eV the saturation current values increase with bias voltage like in the Hecht–Many formula. When we compare the results presented in Figure 12 and Figure 13, taking into account the penetration depth of the radiation, we come to important observations.

We may estimate a possible influence of holes flowing back to the cathode on the kinetics of photocurrent. For photons with energy below *E_g_*, the PC kinetics is very fast, on the order of single seconds, and the hole contribution to the electric current is nearly negligible. However, at high bias voltages, *V* ≥ 100 V, the signal after the first rapid rise (~1 s) increases slightly till about 100 s. This effect we attribute to the contribution of holes to the electric current.

We also investigated samples etched with 10% HCl. The results we obtained by sample excitation with photons of energy of *ħω* = 1.608 eV = *E_g_* + 33 meV (like in Figure 12) as well as those obtained by using energy of *ħω* = 1.531 eV = *E_g_* − 44 meV (like in Figure 13) are identical with the results shown in Figure 13. The etching of the sample with 10% HCl does not generate surface traps.

Thus, we can see that the PC kinetics strongly depends on the surface states and surface charge. The PC-V characteristics at lower voltages and the PC kinetics for *ħω* > *E_g_* provide a method to check for the presence of surface traps.

## 4. Conclusions

At present, photocurrent-voltage characteristics are increasingly used to determine the *µ_e_τ_e_*, which is an essential parameter for X-ray and gamma-ray detectors. The present paper shows PC-V characteristics of semi-insulating (Cd,Mn)Te:In samples with differently prepared surfaces. We show that the different preparations of the surface lead to very different results, and in some cases the Hecht–Many model cannot be applied. Furthermore, the PC-V characteristic features significantly changed with the concentration of HCl used after the polishing process.

We applied the final etching with 20% HCl for some of our samples, measured the PC-V characteristics and fitted our results with the formula given by Ridzonova et al. [13]. The model considers surface states and space charges in the sample volume. We noticed that in the voltage region below saturation, the current is proportional to *V*^3/2^ (in the case of uniform *E* that would correspond to *E*^3/2^). This is in agreement with the Poole–Frenkel model [23,24]. The *I ~ V*^3/2^ dependence is observed even at voltages as low as about 30 V (for our samples with thicknesses ~0.3 cm that would correspond to *E* ~ 100 Vcm^−1^). We have shown that the PC-V method that uses excitation with energy of *ħω > E_g_ + z*, where *z* varies from 20 to 30 meV, is the most suitable for surface effect measurements.

We also noticed that a change in the sample temperature by 4% significantly changed the PC-V characteristic. We roughly estimated that the ionization energy of these surface traps is approximately 0.6 eV below the conduction band. 

We measured the PC kinetics for samples with a high density of surface electron traps. The results obtained by excitation with phonon energy *ħω* *=* 1.608 eV (*E_g_* + 33 meV) differ from those obtained by exciting with *ħω =* 1.531 eV (*E_g_* − 44 meV). Our results show that the measurements of the PC kinetics by using the photons with energies *ħω* *=* 1.608 eV are sensitive to the quality of the surface and may be used to test methods of preparation of detector plates. 

The PC kinetics measured for *ħω* > *E_g_* and *ħω* < *E_g_* indicated a minor influence of holes on the observed phenomena.

We described our results with three models: Hecht [2], Hecht–Many [3] and Ridzonova et al. [13]. The best agreement was obtained for the model by Ridzonova et al. The model considers the non-uniform distribution of the electric field.

We also point out that when the metallic contacts (Au/Pd, Pt/Pd) are deposited directly onto the surface of a high-resistive (Cd,Mn)Te sample with orientation (111), it is better to place the anode on the A (cadmium) side of the plate to obtain high resistivities.

## Figures and Tables

**Table 1:** Parameters obtained from the analysis of the PC-V data for different photon fluxes according to the Ridzonova et al. model [13]: *θ* and *s_e_* are fitting parameters, *N_e_* was determined from the saturation current and *μ_e_τ_e_* was estimated for the lowest photon flux (see text).

LED Current [mA]	Photon Flux *F* [cm^−2^s^−1^] Measured	*θ*	*s_e_* [cm^1^s^−1^]	Photon Flux F* [cm^−2^s^−1^] from Fitting	*N_e_* [cm^−3^]	*μ_e_τ_e_* [cm^2^V^−1^]
0.07	1.39 × 10^12^	5.24 × 10^−4^	4.933 × 10^4^	7.48 × 10^11^	1.7 × 10^6^	7 × 10^−4^
0.13	3.76 × 10^12^	5.25 × 10^−4^	3.685 × 10^4^	1.75 × 10^12^	3.1 × 10^6^	-
0.22	8.49 × 10^12^	5.08 × 10^−4^	4.793 × 10^4^	3.94 × 10^12^	5.0 × 10^6^	-
0.30	1.34 × 10^13^	4.41 × 10^−4^	8.280 × 10^3^	5.84 × 10^12^	6.5 × 10^6^	-
0.45	2.36 × 10^13^	4.67 × 10^−4^	1.038 × 10^4^	1.05 × 10^13^	8.6 × 10^6^	-

## Data Availability

The data presented in this study are available on request from the corresponding author.
